# Recreational fishing, health and well-being: findings from a cross-sectional survey

**DOI:** 10.1080/26395916.2022.2112291

**Published:** 2022-08-31

**Authors:** Pablo Pita, Matthew O. Gribble, Manel Antelo, Gillian Ainsworth, Kieran Hyder, Matilda van den Bosch, Sebastián Villasante

**Affiliations:** a Cross-Research in Environmental Technologies (CRETUS), University of Santiago de Compostela, Santiago de Compostela, A Coruña, Spain; b Campus Do Mar, International Campus of Excellence, Spain; c Gangarosa Department of Environmental Health, Emory University Rollins School of Public Health, Atlanta, GA, USA; d Department of Epidemiology, Emory University Rollins School of Public Health, Atlanta, GA, USA; e Faculty of Economics and Business Administration, University of Santiago de Compostela, Santiago de Compostela, A Coruña, Spain; f Centre for Environment, Fisheries & Aquaculture Science, Suffolk, UK; g School of Environmental Sciences, University of East Anglia, Norwich, UK; h School of Population and Public Health, The University of British Columbia, Vancouver, BC, Canada; i Department of Forest and Conservation Sciences, The University of British Columbia, Vancouver, BC, Canada; j ISGlobal, Parc de Recerca Biomèdica de Barcelona, Barcelona, Spain; k Universitat Pompeu Fabra, Barcelona, Spain; l Centro de Investigación Biomédica en Red Instituto de Salud Carlos III, Madrid, Spain

**Keywords:** Outdoor activities, stress, recreational fisheries, blue and green spaces, public health

## Abstract

We evaluated the associations between marine recreational fishing, stress, seafood consumption, and sleep quality in a cross-sectional questionnaire-based survey of a convenience sample of 244 fishers recruited in 2019 in Spain. Fishers’ stress levels were moderate, with a mean stress index score of 36.4 units on a scale from 14 (very low stress) to 70 (very high). Their average emotional condition was positive, with a mean index of negative affect of 7.8 units on a scale from 5 (very low negative affect) to 25 (very high). Seafood intake was low, with a mean index of seafood in diets of 38.0 units on a scale from 20 (very low seafood consumption) to 160 (very high). Fishers’ perceived quality of night sleep was good because the mean index of sleep problems was 39.5 units on a scale from 21 (very low sleep problems) to 107 (very high). Each hour of self-reported monthly fishing activity was associated with 0.016 units of lower stress score. Thus, the most engaged fishers reported up to 15.4% lower stress score than less avid fishers. Since recreational fishing is a highly accessible outdoor activity for people in older age groups, it is possible that public health could be improved by access to sustainably managed recreational fisheries. Fishing engagement was positively associated with seafood intake. Each hour of fishing per month was associated with one-unit higher seafood consumption. The higher seafood consumption observed among avid recreational fishers compared with less avid fishers might have health implications.

## 1. Introduction

Human populations in urbanized societies tend to carry out daily activities in increasingly denatured habitats, which negatively impacts on how people interact with others and with the environment (Inglehart 1990). The progressive loss of contact with natural environments has been associated with different health problems, mainly triggered by stress (Maller et al. 2006; Bowler et al. 2010). Regular access to natural areas increases people’ś activity, reducing obesity (Björk et al. 2008), and tempers serious health problems related to socioeconomic inequalities, such us e.g. mortality from circulatory diseases, among other (Mitchell and Popham 2008).

Leisure activities can positively influence physical, psychological, and spiritual health (Mannell 2007). Correspondingly, a large number of studies confirm a positive association between exposure and access to natural environments and various health benefits (Hartig et al. 2014; van den Bosch and Sang 2017; White et al. 2019). There is also a wealth of literature describing the positive relationship between human health and recreational activities conducted in natural environments (Mitchell 2013; Chen et al. 2018; Venter et al. 2020), but fishing as a recreational activity is less well explored (Thompson Coon et al. 2011).

We obtained 114 references from a Scopus scientific database search of research papers in English without temporal restrictions by using the following key words: “recreational fisheries” OR “marine recreational fisheries” OR “angling” AND “health” OR “human health”. Once the titles and abstracts of the references were reviewed, it was found that 45 of the reviewed papers referred to a potential association between human health and well-being and recreational fishing. Most of the reviewed scientific literature (36 papers) studied negative effects derived from the consumption of polluted fish by recreational fishers (e.g. Edwards et al. 2001; Pulford et al. 2017; Taylor and Williamson 2017), or injuries and accidents derived from the practice of recreational fishing (Hahn et al. 1993; Read et al. 2017). Only three papers suggested some advantages for people’s health and well-being derived from recreational fishing, including psychosocial effects (Snyder 2007; Griffiths et al. 2016; Young et al. 2016). However, these studies are based on generic perceptions, as in the case of Australian and Solomon Island fishers venting angst and frustration (Young et al. 2016); informed opinion, as in the case of spiritual benefits for fly fishing anglers (Snyder 2007); or hypothetical data (Griffiths et al. 2016). Based on our review, there appear to be no studies that explicitly assess the health and wellness benefits derived from recreational fishing.

Since consuming the catch is a main motivation of recreational fishers (Cooke et al. 2017), in addition to reducing stress and obesity through exercise, another plausible pathway to link the practice of recreational fishing and health benefits relate to seafood consumption. Seafood contains functional components that are not present in most terrestrial organisms. These components include monounsaturated and polyunsaturated fatty acids that have shown protective effects in the prevention of cardiovascular disorders like atherosclerosis, coronary heart, and thrombotic diseases (Lands 1986; Alasalvar et al. 2011; Hosomi et al. 2012; Lund 2013). Moreover, the available evidence provides some support for a benefit of n—23 long-chain polyunsaturated fatty acids to depression illness (Appleton et al. 2010). Preliminary findings also suggest that high levels of omega—3 fatty acids (Murphy et al. 2020) and tryptophan present in seafood could improve sleep quality by increasing melatonin synthesis (St-Onge et al. 2016). Furthermore, seafood is an important source of different nutrients (e.g. selenium and other minerals, amino acids like taurine, peptides and proteins, carotenoids, fiber, and vitamins) that have been related to prevent antioxidative stress and some cancers, to reduce risks of asthma, diabetes, obesity, rheumatoid arthritis, and stroke, and to improve the immune system and the cognitive and visual development (Alasalvar et al. 2011; Hosomi et al. 2012; Lund 2013). Therefore, quantitative assessments of potential health benefits derived from recreational fishing are needed.

Recreational fishing is a popular outdoor activity worldwide, especially in high-income countries where participation rates can reach above 10% of total population (Arlinghaus et al. 2014). Recreational fishers tend to be of relatively advanced age (Steffens and Winkel 2002; Herfaut et al. 2013; Pita et al. 2018), and seafood consumption has been found to increase with age (Olsen 2003). Therefore, finding a link between the practice of recreational fishing and the health and well-being of its practitioners has significant implications for public health in high-income countries with aging populations (Pammolli et al. 2012).

In this study, we evaluated the associations of recreational fishing with major determinants of health and well-being in Galicia (North West Spain) by evaluating three hypotheses: (1) recreational fishing is associated with improvements in the perceived psychological stress of fishers; (2) an increase in fishing effort is associated with more seafood consumption among recreational fishers; and (3) higher seafood consumption among fishers is associated with perceived better quality of night sleep.

## 2. Material and methods

### 2.1. Study design

We performed a cross-sectional study (May – December 2019) targeting a sample of 860 highly engaged marine recreational fishers. They represented 7,122 members of the 30 fishing clubs integrated into the Galician Federation of Responsible Marine Recreational Fishing and Sailing, a private non-profit entity created in 1999 to promote the sustainable practice of recreational fishing and other leisure activities, and the main recreational fishing association in Galicia. The leaders of this fishers’ association and its constituent clubs collaborated in the study from its initial phase to its completion.

In collaboration with the fishers’ association, we organized six events with members of the 30 fishing clubs in key localities around the region where we informed about the objectives of the research and provided ethical and logistical information about participation in the survey. Fishers could collect and complete our anonymous, self-administered questionnaire at these events, or at any time from their club and return them by mail using the stamped addressed envelopes we provided. An incentive program was established consisting of the distribution of merchandise from the project (cotton peak caps) to the attendees, and raffles of fishing gear (fishing rods, reels, and lures) at meetings.

**Figure 1.**
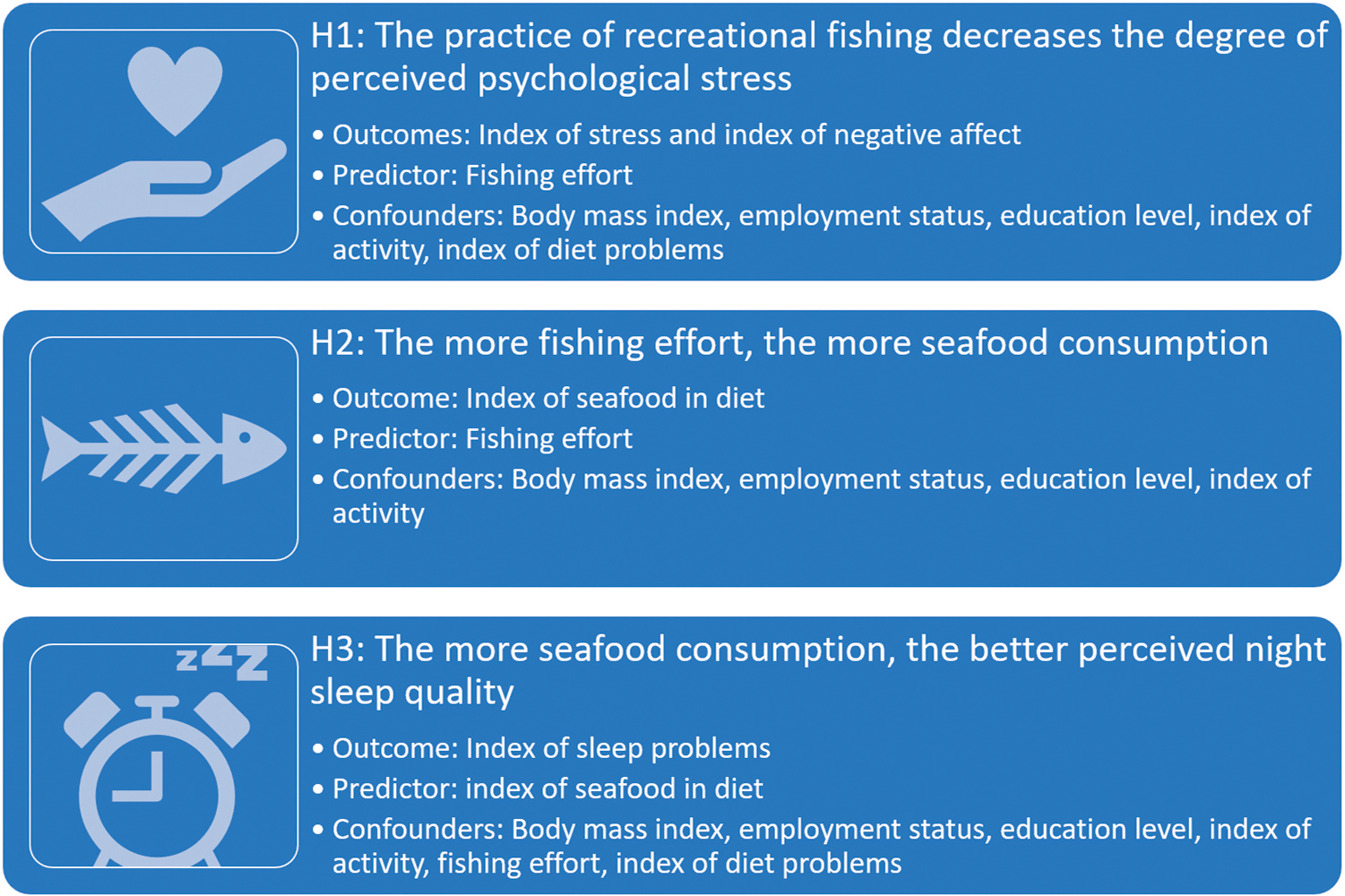
Diagram showing assessed outcomes, predictors, and confounders of the three studied hypotheses.

We designed the questionnaire to obtain information on fishers’ perceived levels of psychological stress, seafood consumption in their diet, and on their perceived quality of night sleep as outcomes of hypotheses 1, 2 and 3, respectively (Figure 1). We obtained information on the degree of recreational fishing effort as a predictor of hypothesis 1 and 2, while degree of seafood consumption was used as a predictor of hypothesis 3 (Figure 1). Anthropometric and socioeconomic data, along with information about other sports and physical activities, and quality of overall diet were used as potential confounders (Figure 1). Additionally, we obtained information on fishers’ ages, fishing platforms (shore or boat), and fishing gears (hook and line or spear gun) to address differences with respect to the total population. For details about the questions see Appendix A of the Supplementary Material. Due to the fact that the participation of women in this fishing community is negligible (less than 1% of fishers are women (Pita et al. 2018)) we did not assess the role of gender in this study. The fishers took 15–30 minutes to complete the questionnaire.

This study followed the Strengthening the Reporting of Observational Studies in Epidemiology (STROBE) guideline for cross-sectional studies (Von Elm et al. 2007) (See Appendix B). The University of Santiago de Compostela (Spain) Bioethics Committee determined that given the use of anonymous data the study was exempt from the need for review and informed consent from participants.

### 2.2. Data processing

#### 2.2.1. Psychological stress

The construct of “psychological stress” is based on a person’s response to the interaction of acute or chronic stressors with the mental structure of the individual and other external modulating factors, such as socioeconomic level (Grzywacz et al. 2004). The stress level can thus be measured by assessing the following two components:

1. Perceived stress, which is the first response to the stressor (Cohen et al. 1983). Several studies have identified this as an important risk factor for the development of many diseases and aggravation of the symptoms of others (Anda et al. 1992; Cohen et al. 1993; Richardson et al. 2012). We measured perceived stress by modifying the 10-question Perceived Stress Scale (PSS) whereby higher scores were associated with greater vulnerability to depressive symptoms and more frequent colds (Cohen et al. 1995). We translated the PSS into Spanish and added four questions to improve ease of understanding of the questionnaire (see Appendix A). Thus, our modified PSS consisted of 14 questions about the previous two-month period with responses graded along a five-point scale (1–5). An index of stress was developed using a scale from 14 (meaning very low stress problems) to 70 (very high stress problems) by reversing response scores following (Cohen et al. 1983) and adding scores together. The mode of each variable of the index was used to fill gaps in the information provided by some of the fishers (14–25 missing data). The value of the Cronbach’s alpha indicated that the index of stress was consistent (α = 0.83; Table 1).
2. Degree of negative affect, which accounts for the affective state characterized by aversive emotional states. Lower levels of negative affect are indicative of a state of calm and serenity, while higher levels indicate a high level of distress (Bolger et al. 1989). We specifically assessed fishers’ feelings of disgust, anger, fear, nervousness, and guilt experienced during the previous month. We adapted five questions using a five-point scale (from 1 to 5) from the cognitive-affective subscale of the Beck Depression Inventory (BDI). The BDI is a 13-item self-report instrument that is highly reliable for gathering information recalled over a one-month period, and designed to assess a wide variety of symptoms and attitudes related to depression (Beck et al. 2000). An index of negative affect was obtained by adding the scores of the five questions scaled from 5 (very low negative affect), to 25 (very high). The mode of each variable of the index was used to fill gaps in the information provided by some of the fishers (19–25 missing data). The internal consistency of the index of negative affect was high (α = 0.80; Table 1).

#### 2.2.2. Fishers’ diet

Overall food patterns, rather than single nutrients, are better to investigate aspects related to food consumption (Hu 2002). The Food Frequency Questionnaire (FFQ) used in this study was a modified version of a questionnaire with 118 food items that has been shown to be a highly reproducible and reliable scale to assess nutrient intake (Martin-Moreno et al. 1993). Our FFQ suits the Spanish cultural background and geographical differences across the country to obtain information on fishers’ complete diet. We asked about the consumption of 97 different food items over the last year (portions, or units; see Appendix A), following the main food categories of the FFQ: drinks, oils, seafood, meat, vegetables, fruits, cereals, dairy, and miscellanea. Responses were graded according to an eight-point scale (1–8). An index of diet problems was obtained by adding the item scores together, scaled from 97 (very low diet problems) to 776 (very high). Potentially healthy (e.g. water) and unhealthy (e.g. energy drinks with caffeine and sugar) food items were included in the index of diet problems in a scale where 1 meant high consumption and 8 meant low consumption. The mode of each variable of the index was used to fill gaps in the information provided by some of the fishers (24–76 missing data). The internal consistency of the index of diet problems was high (α = 0.95; Table 1).

In our FFQ we specifically asked about frequency in the diet of 20 different fish and shellfish species to take into account potential variations in nutritional value (Öhrvik et al. 2012). These 20 questions were used to obtain an index of seafood in diet by adding the scores of the 20 seafood items together, scaled from 20 (very low consumption) to 160 (very high). The mode of each variable of the index was used to fill gaps in the information provided by some of the fishers (28–36 missing data). The internal consistency of the index of seafood in the diet was high (α = 0.94; Table 1).

#### 2.2.3. Night sleep quality

Fishers’ perceived quality of night sleep was assessed through a combination of the perceived degree of satisfaction of night sleep and its duration, established according to the Oviedo Sleep Questionnaire (OSQ), valid to assess insomnia and hypersomnia (Bobes et al. 2000). We expanded the OSQ by adding two questions related to nap habits and use of digital screens before night’s rest, asking 20 questions about the immediate two-month period with responses graded in a five-point scale (1–5) and one in a 1–7 scale. An index of sleep problems was obtained by adding the scores of the 21 items together, scaled from 21 (very low sleep problems) to 107 (very high). The mode of each variable of the index was used to fill gaps in the information provided by some of the fishers (11–29 missing data). The internal consistency of the index of sleep problems was high (α = 0.86; Table 1).

#### 2.2.4. Fishing effort and other physical activities

We obtained the degree of recreational fishing activity (i.e. fishing effort measured in h·month^−1^) during the previous three months. Cases with missing data were excluded from the analyses (N = 15). We also obtained information on the practice of other sports and physical activities as a potential confounder. We implemented a valid and reliable questionnaire developed by Baecke et al. (1982) used in epidemiological studies to measure physical activity in the previous week. We considered all types of habitual physical activity, including work activity, sport, and leisure time, at four levels of intensity, including daytime rest, and frequency of practice. We asked seven questions about the frequency of each of the four intensity levels with responses graded, or converted, using a seven-point scale (1–7) to build an index of activity, scaled from 4 (very low activity) to 154 (very high). Cases with missing data (N = 66) were excluded from the analyses. The internal consistency of the index of activity was acceptable (α = 0.63; Table 1).

#### 2.2.5. Physical and social characteristics

Anthropometric data was obtained regarding age, weight, and height. We calculated the Body Mass Index (BMI) from the latter two. Cases with missing data were excluded from the analyses (N = 7). Additionally, we estimated the socioeconomic status of the fishers by asking about employment status and educational level (Shavers 2007). Cases with missing data were excluded from the analyses (N = 19 and N = 18, respectively).

### 2.3. Statistical analyses

Generalized linear models (GLMs) were used to analyze relationships between the different outcomes, predictors and confounding variables by using the statistical software R ver. 4.0.2 (R Core Team 2019). Continuous potential predictors and confounding variables were transformed into factors by using tertiles and quartiles of each distribution, resulting in variables with three or four levels, respectively.

To test the association between the degree of fishers’ perceived psychological stress and fishing activity (hypothesis 1; Figure 1), fits of fishing effort as predictor of stress and negative affect indexes were obtained from unadjusted models, whereas a backward stepwise selection procedure was followed to fit adjusted models (i.e. from saturated models to final models, removing non-significant variables at each step). The same procedure was followed to assess if seafood intake in fishers’ diets could be predicted by fishing activity (hypothesis 2). In this case the index of seafood in diet was included as an outcome to assess the performance of fishing effort, among other predictors and confounding variables (Figure 1). Finally, the index of sleep problems and the index of seafood in diet were used to assess association between perceived night sleep quality and seafood consumption (hypothesis 3; Figure 1).

Fit of numeric and factorized variables, and that of different error structures and link functions were compared in the different model selection procedures. The best models were selected based on the Akaike’s information criterion (Akaike 1973), goodness of fit (R^2^), and appropriate residual structure. Interactions between different variables and the inclusion of polynomial terms were also assessed. Models with highly dispersed and anomalous distribution of residuals were discarded. Furthermore, our primary analysis for handling missingness would be reasonable under Missing Completely at Random (MCAR) missingness. As a sensitivity analysis for possible departures from MCAR, we used multiple imputation by chained equations (MICE) (Azur et al. 2011) as implemented in the R “mice” library of R (Van Buuren and Groothuis-Oudshoorn 2010). We generated 40 imputed datasets and used the following variables in our imputation model: 14 variables to assess stress, 5 to assess negative affect, 97 variables to assess fishers’ diet, including 20 variables to assess seafood consumption, and 20 variables to assess sleep quality (see Appendix A).

The single role of the different food items to fishers’ perceived night sleep quality was analyzed by a SIMPER procedure (Clarke 1993) included in the “vegan” library of R (Oksanen et al. 2019), performing pairwise comparisons to estimate the average importance of each diet element to the average overall Bray-Curtis dissimilarity.

## 3. Results

We obtained and analyzed 244 questionnaires from recreational fishers, representing 27.9% of the active fishers in the clubs (i.e. 12.1% of fishers belonging to the 30 clubs). Mean age of respondents was 55.0 years (95%CI, 53.2–57.0 years; ranging from 16 to 85), while the mean age of the Galician population is 47.5 years (Xunta de Galicia 2019). Mean BMI of respondents was 27.4 (95%CI, 26.7–28.0), which is equivalent to a weight higher than normal, or overweight. In Galicia, up to 46.7% of population is overweight or obese (Xunta de Galicia 2019). Slightly less than half of the fishers who answered this question (N = 225) were retired (40.0% of answers), while in Galicia 32.1% of the total population is retired (Xunta de Galicia 2019). Most respondents finished high school (37.6%), secondary school (22.6%) or primary school, or obtained a university degree (17.3%, each), while few (5.3%) did not complete any studies (Table 1). In Galicia, 2.1% of people have not completed any study, 42.9% finished primary school, 25.1% finished secondary school, 19.2% finished high school, and 10.7% finished university studies (Xunta de Galicia 2019).

Average fishing effort was 57.9 h·month^−1^ (95%CI, 49.9–67.1 h·month^−1^). Fishers’ preferred fishing from boats (68.8%) or from the shore (45.9%). Their favorite gear was hook and line (97.5%) while some also practiced spear fishing (11.5%). Mean fishing effort was higher than average boat anglers in Galicia (48 h·month^−1^ in summer, the busiest season (Pita et al. 2018)). Moreover, fishers showed a relatively high degree of engagement in other sports and physical activities, since the mean index of activity was 51.1 units (95%CI, 45.7–56.7 units) on a scale from 4 (very low activity rate) to 154 (very high) (Table 1). As a reference, the Galician population spends an average of 43 minutes per day in sports and outdoor activities (Xunta de Galicia 2019), which exceeds the minimum daily recommendation of the World Health Organization (21.4–42.9 minutes per day of moderate-intensity aerobic physical activity) (World Health Organization 2020).

Fishers’ average perceived stress was moderate since the mean index of stress was 36.4 units (95%CI, 35.6–37.3 units) on a scale from 14 (very low stress), to 70 (very high). Similarly, up to 34.8% of the Galician population also felt tense (in the last four weeks) on a regular basis (from sometimes to always) (Xunta de Galicia 2019). On the contrary, fishers’ emotional condition could be considered positive, since the mean index of negative affect was 7.8 units (95%CI, 7.4–8.2 units) on a scale from 5 (very low negative affect) to 25 (very high) (Table 1). Similarly, up to 70.0% of the Galician population never, or rarely felt discouraged and depressed (in the last 4 weeks) (Xunta de Galicia 2019).

Fishers showed moderately unhealthy dietary habits, with a mean index of diet problems of 474.2 units (95%CI, 469.9–478.6 units) on a scale from 97 (very low diet problems) to 776 (very high). Furthermore, seafood intake was relatively low, since the mean index of seafood in the diet was 38.0 units (95%CI, 35.5–40.1 units) on a scale from 20 (very low seafood intake, i.e. less than one ration per month and seafood species) to 160 (very high, i.e. more than three rations per day) (Table 1). In Galicia, 4.3% of the average annual household shopping bag (in weight) corresponds to seafood (30.3 kg·year^−1^) (Gobierno de España 2020).

Finally, fishers’ perceptions about their quality of night sleep were good, since the mean index of sleep problems was 39.5 units (95%CI, 38.2–40.7 units) on a scale from 21 (very low sleep problems) to 107 (very high) (Table 1). The quality of the night’s rest of the general population is also good, since only 22.4% of people in Galicia report difficulty falling asleep on a regular basis (from some nights to every night); up to 60.0% of Galicians never, or rarely awake several times while sleeping; and only 23.6% of population regularly wake up too early (Xunta de Galicia 2019).

### 3.1. Association between fishing effort and psychological stress

**Figure 2.**
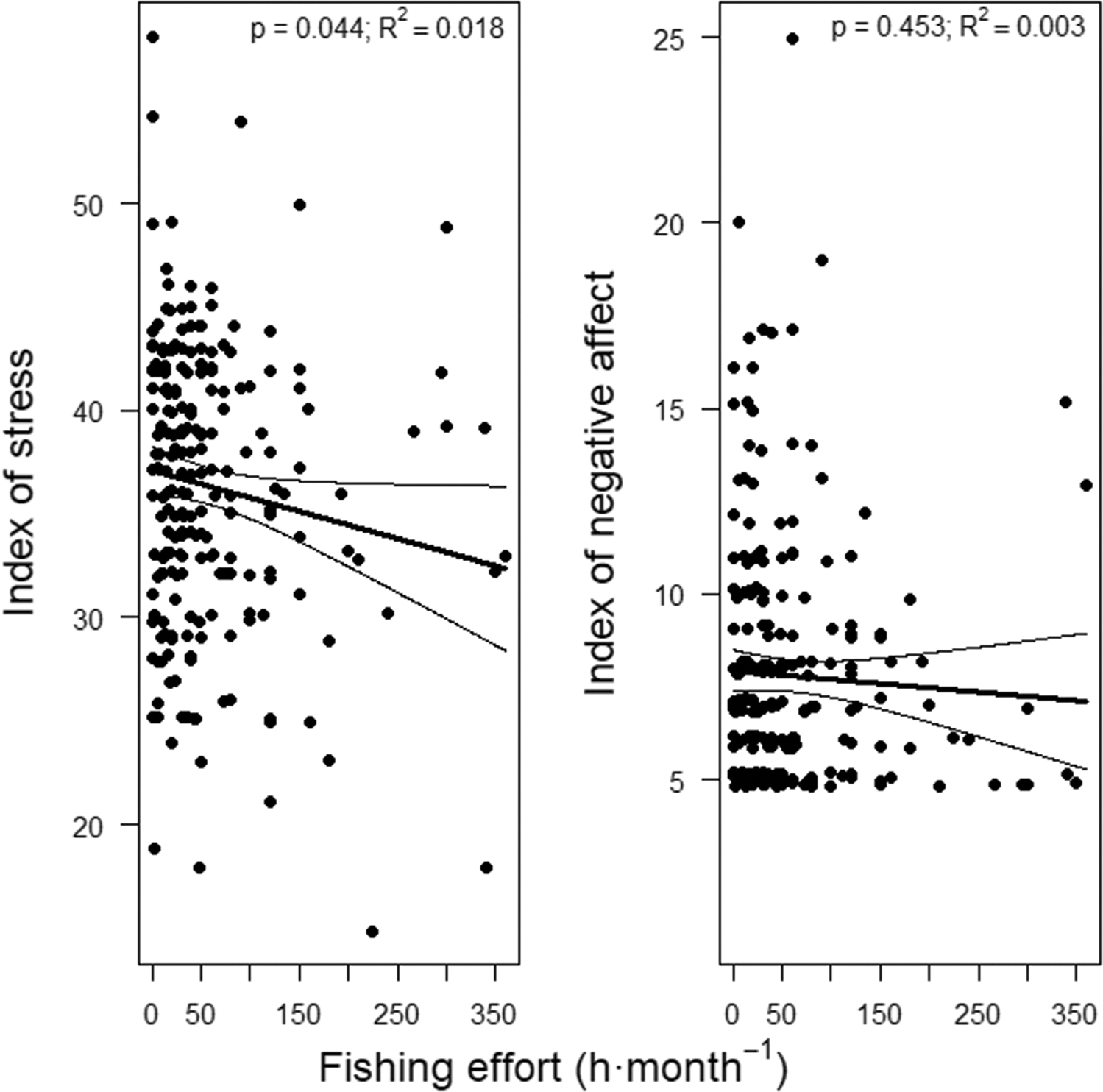
Partial effect of fishing effort on the index of stress and on the index of negative affect of recreational fishers. We show observations (dots), predictions (thick lines) and 95% confidence intervals (thin lines) estimated by unadjusted GLM and TM, respectively. *P*-values, and goodness of fit of the GLM (R^2^) are also shown.

Fishers’ practice of recreational fishing (i.e. fishing effort) was linked to a reduced degree of psychological stress. According to the unadjusted model (p = 0.044; R^2^ = 0.018), for each hour of monthly fishing the index of stress decreased by 0.013 units (95%CI, 0.0–0.026 units) on a scale between 14 (very low stress) and 70 (very high), while perceived stress decreased by 0.016 units (95%CI, 0.003–0.029 units) according to the adjusted model (p = 0.020; R^2^ = 0.045) (Table 2). Thus, the most avid fishers who had fished up to 360 h·month^−1^ in the previous two months reported 12.8% (95%CI, 5.0–21.0%) to 15.4% (95%CI, 7.1–24.2%) lower stress than that reported by fishers that did not go fishing in the same period, according to the unadjusted and adjusted models respectively (Figure 2).

In addition to fishing effort, the adjusted model also showed differences in the index of stress by education (continuous confounder), with lower stress associated with higher education levels (Table 2).

The index of negative affect was not significantly related to fishing effort (p = 0.263; Table 2, Figure 2). Moreover, although none of the adjusted models was significant, retired fishers showed lower negative affect than active fishers in the unadjusted models, while in general lower negative affect tended to be shown by the most physically dynamic people (Table 2).

### 3.2. Association between fishing effort and seafood consumption

**Figure 3.**
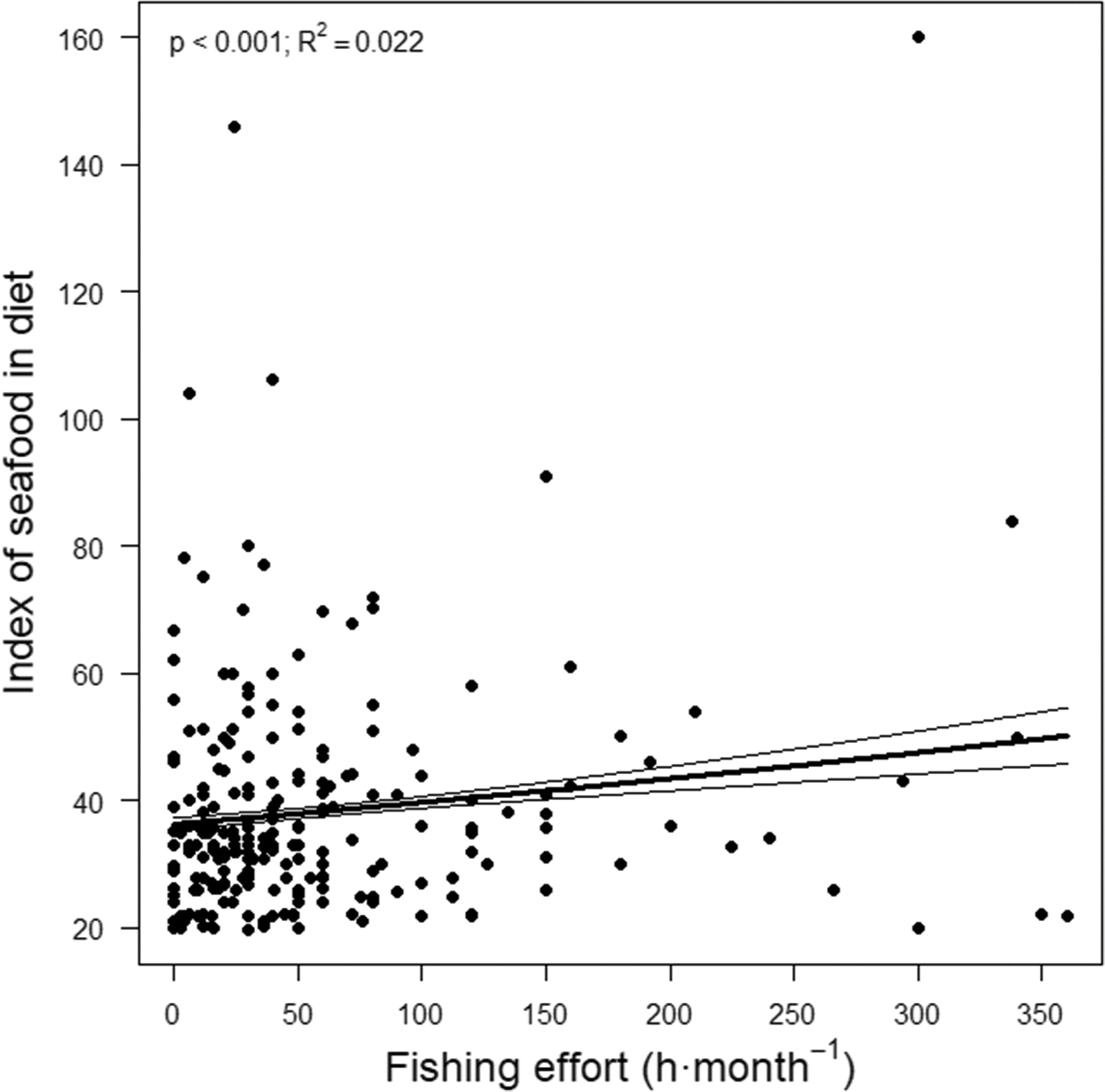
Partial effect of fishing effort on the index of seafood in the diet of recreational fishers. We show observations (dots), predictions (thick lines) and 95% confidence intervals (thin lines) estimated by unadjusted TM. *P*-value is also shown.

The more frequently fishers practiced recreational fishing the higher their intake of seafood (*p* < 0.001; R^2^ = 0.022; Table 2). According to the unadjusted model (none of the adjusted models were significant), for each hour of monthly fishing the index of seafood in diet increased by 1.0 unit (95%CI, 1.0006 1.0012 units) on a scale from 20 (very low seafood consumption, i.e. between one and three rations per month and seafood species) to 160 (very high, i.e. more than three rations per day) (Table 2). Thus, the most avid fishers (360 h·month^−1^ in the last two months) reported on average a 27.6% (95%CI, 23.2–31.9%) higher seafood intake than fishers that did not go fishing (Figure 3). The index of activity also showed a positive association to the index of seafood in diet, with higher levels of physical activity associated with higher seafood consumption (*p* < 0.001; Table 2).

### 3.3. Association between seafood consumption and night sleep

We found no evidence that seafood consumption was associated with perceived sleep quality of recreational fishers. This could be due to the similar frequency of consumption of the different seafood species whereby fishers ate less than one portion per month of all species (mode = 1), except squid and octopus which were eaten one to three times per month (mode = 2). However, we also found no evidence that fishers’ overall diet was associated with perceived quality of night rest. On the other hand, people with higher degrees of physical activity tended to show lower sleeping problems (Table 2).

**Figure 4.**
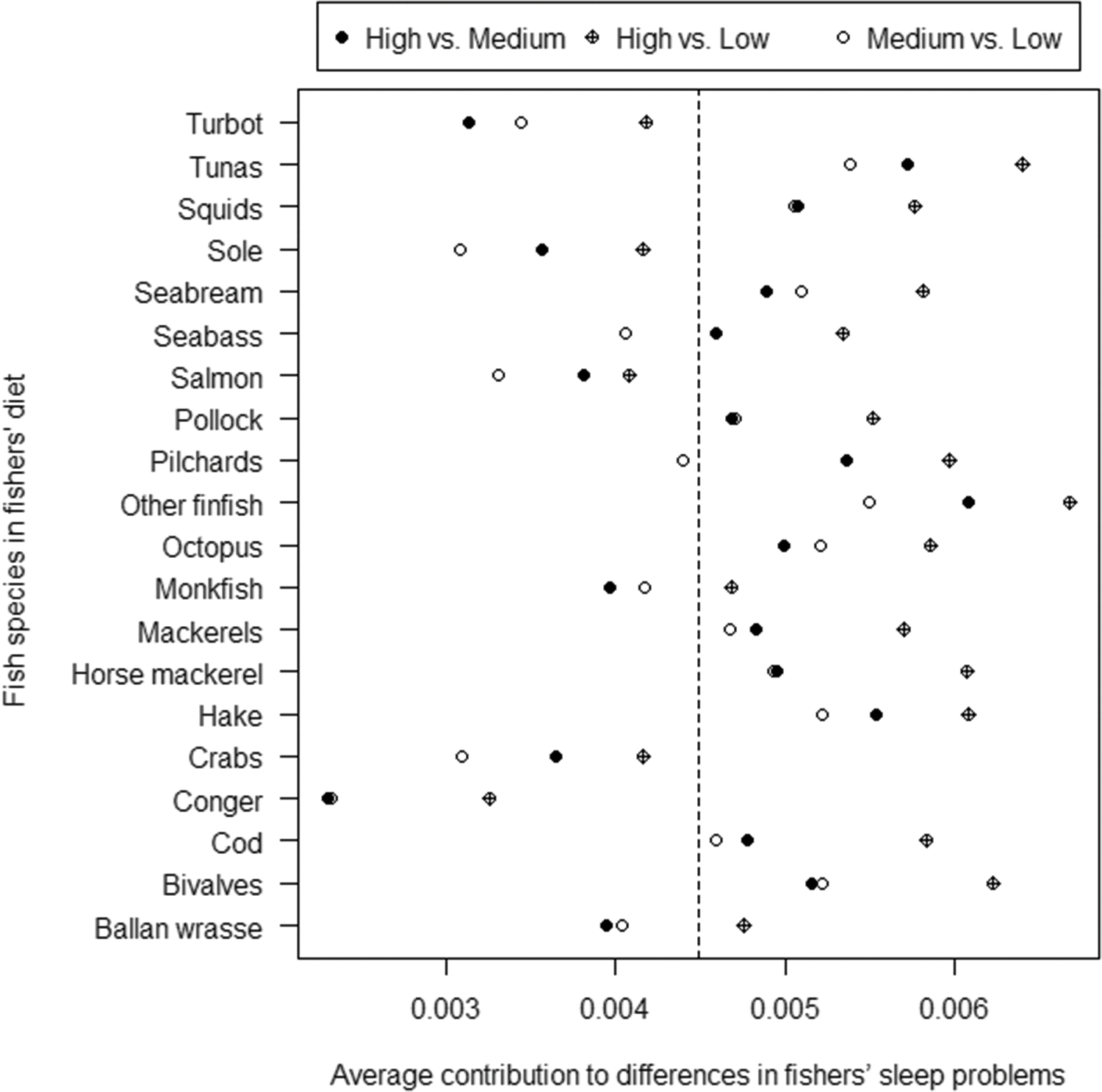
Output of a SIMPER procedure showing the contribution of each seafood species to differences in fishers’ index of sleep problems. The median of the index of sleep problems scores is shown with a dashed line.

The exploratory SIMPER procedure showed some variability in the potential role of the different seafood species to differences in fishers’ perceived night sleep quality i.e. in the categorized index of sleep problems in low (<39), medium (39–49), and high (≥50) (Figure 4). In addition to the generic “other finfish”, the species more relevant to eventual differences in fishers’ perceived sleep quality were tuna, hake, and bivalves (Figure 4).

Notably, the importance of seafood to fishers’ perceived night sleep quality was relatively lower than that of other main food categories. The most important seafood for differences in sleep quality as reported by fishers was the generic “other” when the cumulative dissimilarity achieved approximately half of the total (Table 3).

## 4. Discussion

We demonstrated that the practice of recreational fishing was associated with lower levels of perceived stress in our sample of fishers, but we found no association with negative affect, i.e. aversive emotional states. We also demonstrated that avid recreational fishers showed higher seafood intake in their diets, but no association was found with perceived night sleep quality. In fact, seafood was less important to night rest, compared to other food groups.

### 4.1. Importance

To our knowledge, this study is the first to provide empirical evidence about the benefits for fishers’ health and well-being derived from recreational fishing. Our results are in line with a growing number of studies that describe these types of benefits for practitioners of leisure activities in natural areas (e.g. Mitchell and Popham 2008; Bowler et al. 2010; Hartig et al. 2014), including theoretical and qualitative studies on recreational fisheries (Snyder 2007; Griffiths et al. 2016; Young et al. 2016). We found that perceived psychological stress was lower among retired fishers and decreased with education level, and with the practice of sports and other activities, as stated by other authors (Matthews and Gallo 2011; Santiago et al. 2011; Cohen and Janicki-deverts 2012). However, we found no evidence of an association between perceived psychological stress and diet quality, a relationship that was established in previous studies (Moore and Cunningham 2012).

It has been suggested that the promotion of recreational outdoor activities has collective benefits for society, in the sense of less pressure on public health systems (Maller et al. 2006; van den Bosch and Sang 2017; White et al. 2019). In this regard, recreational fishing is a relatively inexpensive and easily accessible activity for all ages, including older people, since it is not physically demanding (Ainsworth et al. 2000). Therefore, our results may have relevant socioeconomic implications for public health systems, especially in countries with aging populations. In addition to the direct economic benefits derived from recreational fishing for local populations, as recognized by international institutions (e.g. European Parliament 2018), pressure on public health systems could also be relieved by better development of recreational fisheries, among other outdoor activities. For instance, although men and women involved in outdoor consumptive activities like recreational fishing may have different access motivations and expected outcome could vary between sexes (Morales-Nin et al. 2021), gender gap in recreational fishery access, highly mediated by social norms around gender roles (Lee et al. 2009), should be addressed so that more women could enjoy the potential health benefits of recreational fishing.

Contrary to the results obtained by other authors (Peuhkuri et al. 2012; Chaput 2014; St-Onge et al. 2016), we did not observe an association between diet quality and night rest. The relatively low number of observations obtained, among other potential sources of bias that are discussed in the next section, could have masked this association. The fact of not having incorporated factors that could potentially affect the quality of sleep due to the limitations derived from the inclusion of sensitive questions in a questionnaire, already very complex and complete, such as the use of drugs (Vitiello 1997; Wong et al. 2004; Lund et al. 2010), or the type of work (Åkerstedt et al. 2002; Dahlgren et al. 2005; Vahle-Hinz et al. 2014) may also have influenced this output. Furthermore, we found that seafood was less relevant to sleep quality compared with other food groups^1^. The small variability in seafood consumption in our sample may have contributed to the lack of association with nighttime sleep. Temporal variability in exposures and outcomes might also have led to a measurement error that could have caused some attenuation effect. For instance, the supply of some food items, e.g. fruits and vegetables, but also seafood species, typically experience seasonal variations through the year, which could have affected the association with psychological stress, or night’s rest (measured during the last weeks). However, it is expected that the variety of food items in the questionnaire, and seafood (with up to 20 different items), included substitutes with similar market availability, fishing catchability and nutritional properties.

Moreover, the so-called “ecological fallacy” (Piantadosi et al. 1988) could have been problematic if the pattern observed at the larger aggregated unit (one year in the case of associations with diet) was not equivalent to what would be observed at the smaller unit (last weeks in the case of psychological stress, or night’s rest). In fact, it cannot be ruled out that eventual short-term benefits (e.g. for night’s rest after consuming seafood during the last weeks) change with time and even decline because of trade-offs (e.g. toxic contents in flesh). It is necessary to take into account that some fish and shellfish contain elements such as heavy metals (Blanco et al. 2008) among other contaminants that could counterbalance the potential benefits of a diet rich in seafood (Rodríguez-Hernández et al. 2016), including night’s rest (Arito et al. 1983; Parmalee and Aschner 2017).

Moreover, seafood are often misidentified in Spain (Horreo et al. 2019) which makes seafood-health associations challenging to interpret given the differences between species in nutrient and contaminant content (Gribble et al. 2016). Tunas, hake, and bivalves were likely to be relatively more important than other seafood species in relation to perceived sleep quality. Notably, dishes cooked with these ingredients have, in previous research, shown very high levels of tryptophan content (Öhrvik et al. 2012). Therefore, in accordance with other studies (Hansen et al. 2014; Del Brutto et al. 2016; Liu et al. 2017), benefits derived from diets rich in seafood for the quality of night rest should not be disregarded. In addition to the association between fishing engagement and seafood consumption demonstrated in this study, recreational fishers buy more expensive, high-quality fresh fish in local markets than most people (Morales-Nin et al. 2013). This has major implications for peoples’ health in a context of epidemic obesity because the trend towards progressively impoverished diet habits (Kosti and Panagiotakos 2006) exists in countries with higher seafood consumption such as Japan, Portugal and Spain (FAO 2020).

### 4.2. Limitations

#### 4.2.1. Convenience sampling and lack of representativeness

Even though non-randomized sampling, including self-administrated questionnaires like the one used in our study, are less time and money consuming than other methods, they may have limitations in terms of their representativeness if some members of the population are less likely to be included than others, and therefore the sample is not representative of the overall population (Venes 2017). However, immigrative selection bias is a problem when the participation is affected by both the exposure and the outcome, which is not clear in the associations studied here.

As a general reference, all the demographic variables measured in our sample (mean age, BMI, employment situation, and study level), and most of the variables related to health and well-being (mean physical activity, stress, negative affect, and quality of night sleep) did not differ from the average data available for the Galician population. Nevertheless, it is possible that the fishers who participated in this study show higher consumption of seafood per capita (about 45 kg·year^−1^) than the Galician average (30 kg·year^−1^). The consumption of their own catches, and a higher demand for marketed fresh seafood shown by recreational fishers in Spain (Morales-Nin et al. 2013) could explain this difference.

Differences in age, fishing platform and gear, and in fishers’ avidity (i.e. fishing engagement, measured as fishing effort) between the sample and the studied population are relevant potential sources of bias in recreational fisheries research (Pollock et al. 1994). Socioeconomic profiles of the fishers who participated in our study (e.g. employment status and education level) were similar to those shown by Pita et al. (2018) regarding all Galician recreational fishers. However, the mean age of fishers in this study (55 years) was more similar to that of boat anglers in Galicia (53 years), than to that of Galician shore anglers (50 years), and spear fishers (37 years) (Pita et al. 2018). In fact, despite shore anglers being the most common type of recreational fishers in Galicia (75% of total), followed by boat anglers (20%) and spear fishers (5%) (Pita et al. 2018), they participated less in this study (46% of the sample), compared to boat anglers (69%) and spear fishers (13%). Furthermore, since we specifically targeted the most active fishers in the clubs, we anticipated a greater presence of avid fishers in our study. As expected, fishers showed higher mean fishing effort in this study (58 h·month^−1^) than the average recreational fisher in Galicia (up to 48 h·month^−1^ in the case of boat anglers during summer which is the busiest season (Pita et al. 2018). Consequently, our results might present a higher degree of uncertainty for less avid fishers, and especially for shore anglers.

Moreover, we measured perceived stress, diet, sleep quality and physical activities with instruments designed for other populations. Although this could be problematic in some cases, we expect enough commonalities with our studied population to be applied without being a relevant source of bias.

#### 4.2.2. Non-response bias

Non-response bias affects surveys when certain groups of fishers refuse to participate in the survey, or do not answer specific questions, affecting the representativeness of the study (Fisher 1996). We regard our sample as a convenience sample rather than representative due to the low response rate (McColl and Thomas 2000). Despite efforts made to publicize this study throughout the Galician fishing clubs, it is likely that many fishers were not aware of it because face-to-face social activity in the Galician clubs is relatively limited. Difficulties in completing a long and detailed questionnaire may also have discouraged some fishers. No trends were detected regarding the number of responses to the different questions. Therefore, MCAR assumptions regarding the procedure that we followed to impute missing data are reasonable (see Appendix C). However, it cannot be ruled out that certain groups of fishers have refused to participate in the study, e.g. because of mistrust of researchers and fisheries managers after unsatisfactory relationships in the past (Pita and Villasante 2019; Pita et al. 2020). It is difficult to estimate the extent to which our results have been affected by an eventual lack of response from specific groups of fishers.

#### 4.2.3. Recall bias

Fishers tend to overestimate their effort, including frequency of participation (Thompson and Hubert 1990), when the recall period exceeds several months (Hiett and Worrall 1977; Pollock et al. 1994). Since our recall period was the previous three months it is not expected that the fishing effort has been overestimated. Nevertheless, recall bias might have affected some of the responses given regarding fishers’ perceived psychological stress because mechanisms that modulate memories of past emotional states remain unclear (Colombo et al. 2020), while some uncertainty about how to assess outcome scale responsiveness could remain (Robling and Hood 2002). Overall diet and seafood intake could also have been affected by long-term memory limitations. However, although recall bias could have increased errors within the perceived stress and diet-related variables, making it difficult to obtain statistically significant results in the models, we do not expect trends in the responses that could lead to spurious relationships.

#### 4.2.4. Declaration bias

Declaration bias can occur when some respondents answer some questions idiosyncratically according to their convenience (Pollock et al. 1994). However, we do not expect that the fishers have foreseen the data treatment that would be conducted on their question responses, so we assume this bias has not affected the results. In addition, given that we used the same questionnaire to collect the data used to construct the predictor variables, we do not rule out some degree of regression attenuation derived from errors in obtaining the information, which would have impaired our ability to obtain significant results, e.g. in the case of the association between sleep quality and seafood consumption.

#### 4.2.5. Reverse causation

We do not rule out the possibility that fishers with lower levels of psychological stress go out fishing more. However, although other studies should be carried out to confirm our hypothesis, the fact that the sampled fishers are themselves a group highly involved in fishing suggests that this leisure activity has a positive effect on their stress levels.

#### 4.2.6. Other limitations

Recreational fishers show great heterogeneity in terms of their motivations for accessing and staying in the fishery (Knoche and Lupi 2016; Magee et al. 2018; Matsumura et al. 2019). This diversity also affects the frequency and duration of fishing trips (Dabrowksa et al. 2017). Even though in Galicia access to fishing areas is relatively quick and easy for the entire population, fishers living closer to the coast may show higher fishing effort. Although the lack of this information in the models did not bias the results, including proximity to fishing areas could improve the output of the models that included fishing effort as predictor. Similarly, although the influence of the age of the fishers in the different models should have been largely captured by the employment status, its inclusion could have contributed to better fits.

This is an observational study that may not have accounted for all possible confounders. Since social interactions are important for certain profiles of recreational fishers (Mueller et al. 2008), including Galician fishers (Pita et al. 2018), the role of the social network structure may have been important. It is possible that fishers with stronger social networks, with greater chances to go fishing with someone else, also show a better state of mind derived from the protective role offered by social integration (Cohen and Lemay 2007; Rosenquist et al. 2011). Similarly, social networks can also influence dietary choices (Pachucki et al. 2011) and sleep quality (Gordon et al. 2017).

## 5. Conclusions

The association between the practice of marine recreational fishing and benefits for the health and well-being of people demonstrated in this study constitutes the basis for further research on this topic, which must necessarily be developed in order to begin to reverse the notable lack of scientific attention to the social benefits of an outdoor activity that is practiced by almost 60 million people and generates about 30 billion dollars annually worldwide through the expenses and investments of the fishers (Cisneros-Montemayor and Sumaila 2010). Since the relationship between human health and recreational fishing has relevant implications for public health systems, especially in developed countries with aging populations, it is recommended that future studies confirm a causal relationship and minimize representativeness biases with respect to the general fishers’ population. Randomized clinical trials would be optimal, but other observational research designs like cohort, or case-control would also be suitable if confounders and selection bias are under control (Mann 2003; Mariani and Pego-Fernandes 2014).

It would also be advisable to keep under control the heterogeneity of fishers in future studies, e.g. in terms of their access motivations (Fedler and Ditton 1994), orientation toward catches (Beardmore et al. 2011), fishing skills (Scott and Shafer 2001), or the importance of fishing for their lifestyle (Kyle et al. 2007). These dimensions can help to understand how different groups of recreational fishers respond to external events (see e.g. Pita et al. 2021). Including the proximity to coastal fishing areas, and some information on the social network structure could also help to improve the model outputs.

Although we were unable to identify a relationship between seafood consumption and sleep quality, the association between fishing intensity and seafood consumption demonstrated in our study, and the accumulated scientific evidence on the positive effects of diets rich in seafood on different aspects of health (Appleton et al. 2010; Hosomi et al. 2012; Lund 2013), including night rest (St-Onge et al. 2016; Murphy et al. 2020), lead us to recommend including dietary aspects in subsequent research initiatives.

## Supplementary Material

Appendix B

Appendix C

Appendix A

## Figures and Tables

**Table 1.** Physical and socioeconomic characteristics of the participants, and results of the indices obtained from fishers’ responses to a self-administrated questionnaire. We show the Cronbach’s alpha (α) of the indices.

Item	N	Mean	95%CI	α

Mean age	235	55.00	53.20–57.0	
Body Mass Index	237	27.43	26.69–28.01	
Education				
*Any*	12			
*Primary*	39			
*Secondary*	51			
*High*	85			
*University*	39			
Employment status				
*Active*	135			
*Retired*	90			
Fishing effort	229	57.93	49.88–67.09	
Fishing gear				
*Hook and line*	238			
*Spear gun*	28			
Fishing platform				
*Boat*	168			
*Shore*	112			
Index of activity	178	51.08	45.68–56.65	0.63
Index of diet problems	244	474.15	469.90–478.70	0.95
Index of negative mood	244	7.82	7.37–8.23	0.80
Index of seafood in diet	244	38.00	35.49–40.08	0.94
Index of sleep problems	244	47.09	45.53–48.75	0.86
Index of stress	244	36.37	35.55–37.28	0.83

**Table 2.** Outputs of the GLM and TM fitted on the index of stress, index of negative affect, index of seafood in diet, and index of sleep problems. We show the estimated model coefficients, *p*-values and 95% confidence intervals for the different predictors of unadjusted, and of final adjusted models when available, and of significant confounders. The error and link structure, values of Akaike’s information criterion (AIC), and goodness of fit (R^2^) when possible, are also given (Q=quartile-factorized variable).

Outcome	Predictor/confounder	Class	Family	Intercept	Coefficient	P value	Confidence interval (95%)	Goodness of fit (R^2^)	AIC

Index of stress	Fishing effort	GLM	Gaussian	37.0990	−0.0132	**0.0442**	**−0.0259, −0.0004**	0.0177	1527
Index of stress	Fishing effort	GLM	Gaussian	40.4898	−0.0156	**0.0202**	**−0.0286, −0.0025**	0.0455	1448
	Education				−0.9951	**0.0156**	**−1.7955, −0.1948**		
Index of negative affect	Fishing effort	TM	Gaussian	7.2428	−0.0051	0.2630	−0.0140, 0.0038		1053
Index of negative affect	Employment status: active vs. retired	TM	Gaussian	7.5785	−1.3360	**0.0217**	**−2.4767, −0.1953**		1027
Index of negative affect	Index of activity (Q): very low vs. low	TM	Gaussian	8.2272	−0.7645	0.3879	−2.4998, 0.9708		849
	Index of activity (Q): very low vs. high				−0.3287	0.7102	−2.0628, 1.4053		
	Index of activity (Q): very low vs. very high				−2.0874	**0.0222**	**−3.8764, −0.2983**		
	Index of activity (Q): low vs. high				0.4358	0.6240	−1.3055, 2.1770		
	Index of activity (Q): low vs. very high				−1.3229	0.1480	−3.1167, 0.4709		
	Index of activity (Q): high vs. very high				−1.7586	0.0549	−3.5542, 0.0369		
Index of seafood in diet	Fishing effort	TM	Gaussian	35.6220	0.0394	**0.0340**	**0.0030, 0.0758**		1936
Index of seafood in diet	Index of activity	TM	Gaussian	30.2225	0.1552	**0.0001**	**0.0779, 0.2326**		1492
Index of sleep problems	Index of activity (Q): very low vs. low	GLM	Gamma	42.8890	−3.0890	0.1644	−7.4246, 1.2468	0.0344	1311
	Index of activity (Q): very low vs. high				−5.0480	**0.0213**	**−9.3073, −0.7887**		
	Index of activity (Q): very low vs. very high				−3.3660	0.1307	−7.7104, 0.9780		
	Index of activity (Q): low vs. high				−1.9591	0.3490	−6.0504, 2.1322		
	Index of activity (Q): low vs. very high				−0.2773	0.8970	−4.4569, 3.9024		
	Index of activity (Q): high vs. very high				1.6820	0.4226	−2.4186, 5.7822		

**Table 3.** Output of a SIMPER procedure showing the average contribution of each food item of diet to differences in the tertile-factorized index of sleep problems (s=with sugar; c=with caffeine; sq=squeezed). The different food items have been ordered based on their average relative contribution.

		Index of sleep problems		
Food item		High vs. medium	High vs. low	Medium vs. low

Drinks	Coffee	0.0031	0.0033	0.0032
	Water	0.0028	0.0030	0.0030
	Citrus juices (sq)	0.0025	0.0023	0.0021
	Juices (sq)	0.0023	0.0020	0.0016
	Tea	0.0021	0.0018	0.0016
	Cola (c,s)	0.0017	0.0019	0.0013
	Chocolate drinks	0.0014	0.0014	0.0009
	Cola (c)	0.0013	0.0012	0.0009
	Citrus juices	0.0014	0.0011	0.0008
	Sodas	0.0011	0.0011	0.0009
	Sodas (s)	0.0011	0.0010	0.0007
	Wine (red)	0.0006	0.0007	0.0006
	Energy drinks (c,s)	0.0007	0.0006	0.0004
	Beer	0.0006	0.0007	0.0004
	Energy drinks (s)	0.0005	0.0004	0.0005
	Spirits	0.0002	0.0001	0.0002
	Wine (white)	0.0001	0.0001	0.0001
Oils	Oil for cooking	0.0024	0.0026	0.0026
	Olive oil	0.0022	0.0025	0.0023
	Other oils	0.0024	0.0025	0.0019
	Other oils for cooking	0.0020	0.0020	0.0015
Seafood	Other finfish	0.0018	0.0020	0.0015
	Tunas	0.0017	0.0018	0.0015
	Hake	0.0016	0.0017	0.0015
	Bivalves	0.0015	0.0017	0.0015
	Octopus	0.0014	0.0017	0.0015
	Horse mackerel	0.0014	0.0017	0.0014
	Squids	0.0015	0.0016	0.0014
	Seabream	0.0014	0.0016	0.0014
	Pilchards	0.0015	0.0016	0.0013
	Mackerels	0.0014	0.0016	0.0013
	Cod	0.0014	0.0015	0.0013
	Pollock	0.0013	0.0015	0.0013
	Seabass	0.0013	0.0015	0.0012
	Monkfish	0.0011	0.0013	0.0012
	Ballan wrasse	0.0011	0.0012	0.0012
	Salmon	0.0011	0.0011	0.0009
	Crabs	0.0010	0.0012	0.0008
	Sole	0.0009	0.0012	0.0009
	Turbot	0.0009	0.0011	0.0010
	Conger	0.0006	0.0008	0.0006
Meat	Ham	0.0019	0.0021	0.0018
	Sausages	0.0020	0.0021	0.0016
	Chicken	0.0016	0.0019	0.0016
	Pork	0.0017	0.0019	0.0014
	Cow	0.0015	0.0018	0.0015
	Bacon	0.0013	0.0015	0.0009
	Burger	0.0012	0.0014	0.0009
	Liver	0.0010	0.0012	0.0007
	Frankfurts	0.0011	0.0012	0.0006
Vegetables	Other vegetables	0.0021	0.0022	0.0020
	Onion	0.0021	0.0022	0.0018
	Potatoes	0.0019	0.0021	0.0018
	Tomato	0.0020	0.0020	0.0018
	Carrot	0.0020	0.0020	0.0017
	Lettuce	0.0020	0.0020	0.0017
	Cabbage	0.0019	0.0020	0.0017
	Peppers	0.0018	0.0019	0.0016
	Mushrooms	0.0018	0.0019	0.0015
	Lentils	0.0017	0.0018	0.0015
	Spinach	0.0017	0.0017	0.0015
	Zucchini	0.0017	0.0016	0.0016
	Asparagus	0.0017	0.0017	0.0015
	Peas	0.0016	0.0016	0.0014
	Beans	0.0016	0.0016	0.0013
	Chickpeas	0.0015	0.0016	0.0014
	Eggplant	0.0014	0.0013	0.0012
Fruits	Orange	0.0025	0.0025	0.0024
	Banana	0.0025	0.0026	0.0023
	Apple	0.0022	0.0023	0.0021
	Kiwi	0.0019	0.0020	0.0018
	Peaches	0.0019	0.0020	0.0017
	Melon	0.0019	0.0020	0.0016
	Strawberries	0.0018	0.0019	0.0015
	Pineapple	0.0017	0.0017	0.0016
	Olives	0.0015	0.0016	0.0014
	Berries	0.0016	0.0016	0.0012
	Avocado	0.0013	0.0014	0.0011
Cereals	White bread	0.0025	0.0026	0.0026
	Whole bread	0.0022	0.0022	0.0022
	Breakfast cereals	0.0022	0.0021	0.0020
	Pasta	0.0018	0.0020	0.0016
Dairy	Yogurt	0.0026	0.0027	0.0026
	Milk	0.0023	0.0024	0.0022
	White cheese	0.0021	0.0023	0.0020
	Other cheese	0.0020	0.0022	0.0019
	Butter	0.0014	0.0014	0.0011
Miscellanea	Sugar	0.0027	0.0028	0.0026
	Nuts	0.0023	0.0023	0.0018
	Cookies	0.0020	0.0020	0.0012
	Chocolate	0.0017	0.0017	0.0015
	Eggs	0.0016	0.0017	0.0015
	Baked goods	0.0015	0.0015	0.0010
	Cakes	0.0015	0.0014	0.0010
	Fritters	0.0014	0.0014	0.0009
	Mayonnaise	0.0014	0.0014	0.0010
	Bakery	0.0013	0.0013	0.0008

## Footnotes

1. Seafood is by far the main source of Omega—3 fatty acids (Tur et al. 2012), however tryptophan is also present in milk, meat, poultry, eggs, beans, peanuts, cheese, and leafy green vegetables (Halson 2008).
