# Supplementary material for: Recreational fishing, health and well-being: findings from a cross-sectional survey
Source: Ecosyst People (Abingdon). Author manuscript; Available in PMC 2022 Dec 19. (PMC9762678; doi:10.1080/26395916.2022.2112291)
Supplement: Appendix C [file NIHMS1837679-supplement-Appendix_C.pdf]

Appendix C: Outputs of the GLM fitted on the index of stress, index of negative affect, index of seafood in diet, and index of sleep problems, after performing a multiple imputation procedure of missing values (see text in the Statistical analyses section for further information). We show the estimated model coefficients and p-values for the different predictors of unadjusted, and of final adjusted models when available, and of significant confounders in the case of variables that included missing data. The error and link structure are also given (Q=quartile-factorized variable).

Outcome	Predictor/confounder	Class	Family	Coefficient	P value
Index of stress	Fishing effort	GLM	Gaussian	-0.0131	0.0701
Index of stress	Fishing effort	GLM	Gaussian	-0.0159	**0.0320**
	Education			-0.1913	**0.0039**
Index of negative affect	Fishing effort	GLM	Gaussian	-0.0009	0.7910
Index of negative affect	Employment status: active vs. retired	GLM	Gaussian	-0.8754	0.0730
Index of negative affect	Index of activity (Q): very low vs. low	GLM	Gaussian	-0.6740	0.3147
	Index of activity (Q): very low vs. high			-0.3776	0.5579
	Index of activity (Q): very low vs. very high			-1.3537	**0.0226**
	Index of activity (Q): low vs. high			0.2551	0.7234
	Index of activity (Q): low vs. very high			-0.5813	0.3958
	Index of activity (Q): high vs. very high			-0.8364	0.2354
Index of seafood in diet	Fishing effort	GLM	Gaussian	0.0456	**0.0094**
Index of seafood in diet	Index of activity	GLM	Gaussian	0.0711	0.0962
Index of sleep problems	Index of activity (Q): very low vs. low	GLM	Gamma	-3.6599	0.1453
	Index of activity (Q): very low vs. high			-7.8504	**0.0016**
	Index of activity (Q): very low vs. very high			-1.8654	0.4335
	Index of activity (Q): low vs. high			-3.7862	0.1582
	Index of activity (Q): low vs. very high			-0.0089	0.9975
	Index of activity (Q): high vs. very high			3.7773	0.1660
