# Supplementary material for: Recreational fishing, health and well-being: findings from a cross-sectional survey
Source: Ecosyst People (Abingdon). Author manuscript; Available in PMC 2022 Dec 19. (PMC9762678; doi:10.1080/26395916.2022.2112291)
Supplement: Appendix A [file NIHMS1837679-supplement-Appendix_A.pdf]

Appendix A: Questionnaire to recreational fishers

A. PERSONAL INFORMATION

Please write your date of birth
Please write weight (kg)
Please write height (cm)
Please indicate whether you are active, or retired

B. SOCIOECONOMIC INFORMATION

Please indicate your finished studies (please select: I do not have, primary, secondary, high school, or university)

C. FISHING ACTIVITY

Fishing modality that you usually practice (please select: boat angling, shore angling, or spear fishing)
Type of gears that you usually use to fish (please select: rod and line, hand line, or spear gun)
Please write the number of days you went fishing in the last 3 months
Please write the average number of hours of each fishing trip

D. SLEEP QUALITY IN THE LAST 2 MONTHS

Please write the number of hours that you used to sleeping at night
How satisfied were you with the quality of your sleep? (please select: nothing, very few, few, moderate, satisfied, or a lot)
How long did it take you to fall asleep when you tried? (please select: 0-15 min, 16-30 min, 31-45 min, 46-60 min, or >1 h)
How many times did you wake up at night? (please select: 0, 1, 2, 3, or >3)
How long did you wake up earlier than usual? (please select: as usual, 30 min, 1 h, 1-2 h, or >2 h)
What percentage of the time in bed you were sleeping? (please select: 91-100, 81-90, 71-80, 61-70, or ≤60)

How many days per week ... (please select in each case: 0, 1-2, 3, 4-5, or 6-7)
… it was difficult falling asleep?
… it was difficult staying asleep?
… it was difficult to achieve a restful sleep?
… it was difficult to wake up at the usual time?
… you felt excessive sleepiness?
… you got more worried or tired or had less performance due to not having a good night's sleep?
… you were drowsy and slept during the day or slept more than usual at night?
… you had snoring?
… you had snoring with chocking?
… you had excessive leg movement?
… you had nightmares?
… you had other events during sleep?

If you have been drowsy, how many days did you worry or notice a lower social and labor performance because of it? (please select in each case: 0, 1-2, 3, 4-5, or 6-7)

How many minutes did you get used to napping? (Please select: 0, 1-15, 16-30, 31-45, or ≤45)

Report the time you look at a bright screen just before going to sleep (phone, tablet, computer ...) (please select: rarely, 15 min, 30 min, 45 min, or >45 min)

D. IN THE LAST 2 MONTHS YOU FELT… (please select in each case: nothing at all, a bit, moderately, quite, a lot)
… upset by something that happened unexpectedly?
… unable to control important things in your life?
… nervous or stressed?
…that successfully dealt with life problems?
…that faced important changes in your life well?
… that endured important changes in life well?
… that things are going your way?
…that couldn't cope with things?
… able to put up with irritable issues of life?
… that you were above things?
… angry because things were out of control?

… thinking of things that you had to do?
… you were able to control how you spend your time?
… that the difficulties piled up so much that you were not able to overcome them?

E. IN THE LAST MONTH YOU FELT… (please select in each case: nothing at all, a bit, moderately, quite, a lot)
… sad
… angry
… fearful
… uneasy
... guilty

F. PHYSICAL ACTIVITY IN THE LAST 7 DAYS

How many days did you spend doing intense exercise (the one that makes you breathe much more intensely than normal and for more than 10 minutes)? (please select: 1, 2, 3, 4, 5, 6, or 7)
Report time spent on average each day (please select: 15 min, 30 min, 45 min, 1 h, 1 h 15 min, 1h 30 min, more time)

How many days did you spend doing moderate exercise (carry weights, ride a bicycle at a regular speed. Do not include walking)? (please select: 1, 2, 3, 4, 5, 6, or 7)
Report time spent on average each day (please select: 15 min, 30 min, 45 min, 1 h, 1 h 15 min, 1h 30 min, more time)

How many days did you walk at least 10 minutes? (please select: 1, 2, 3, 4, 5, 6, or 7)
Report time spent on average each day (please select: 15 min, 30 min, 45 min, 1 h, 1 h 15 min, 1h 30 min, more time)

Please write how many hours on average do you spend each day sitting and/or lying on a couch

G. HABITUAL FOOD CONSUMPTION

Soft drinks and drinks (please select in each case: <1/month, 1-3/month, 1/ week, 2-4/week, 5-6/week, 1/day, 2-3/day, or >3/day)
Caffeine-free sodas with sugar
Caffeine-sugar-free sodas
Energy drinks with caffeine and sugar
Caffeine-free energy drinks
Squeezed citrus juices (fresh or packed)
Other freshly squeezed juices
Citrus juice from concentrate (packed)
Chocolate drinks
Tea, tea drinks
Coffee (not decaf, with or without milk)

Water (please write the number of glasses per day)

Alcohol (please write the number of glasses per day/week)
Red wine
White wine
Spirits (alone or with soft drinks)

Oils (please select in each case: <1/month, 1-3/month, 1/ week, 2-4/week, 5-6/week, 1/day, 2-3/day, or >3/day)
Olive oil for cooking or dressing (times of use)
Other oil for cooking or dressing (times of use)
Meals cooked with olive oil (rations)
Meals cooked with other oil (rations)

Seafood (please select in each case: <1/month, 1-3/month, 1/ week, 2-4/week, 5-6/week, 1/day, 2-3/day, or >3/day)
Ballan wrasse (ration)
Cod (ration)
Conger (ration)
Hake (ration)
Horse mackerel (ration)
Mackerels (ration)
Monkfish (ration)
Pilchards (2-3 units)
Pollock (ration)

Salmon (ration)
Seabass (ration)
Seabream (ration)
Sole (ration)
Tunas (ration)
Turbot (ration)
Other finfish (ration)
Bivalves (ration)
Crabs (ration)
Octopus (ration)
Squids (ration)

Meat (please select in each case: <1/month, 1-3/month, 1/ week, 2-4/week, 5-6/week, 1/day, 2-3/day, or >3/day)
Bacon (ration)
Burger (1 unit)
Chicken (ration)
Cow (ration)
Frankfurt (2)
Ham (ration)
Liver (ration)
Pork (ration)
Sausages (ration)

Vegetables (please select in each case: <1/month, 1-3/month, 1/ week, 2-4/week, 5-6/week, 1/day, 2-3/day, or >3/day)
Asparagus (1 unit)
Beans (ration)
Cabbage (ration)
Carrot (1 unit)
Chickpeas (ration)
Eggplant (half)
Lentils (ration)
Lettuce (ration)
Mushrooms (ration)
Onion (half)
Peas (ration)
Peppers (ration)
Potatoes (ration)
Spinach (ration)
Tomato (ration)
Zucchini (ration)
Other vegetables (ration)

Fruits (please select in each case: <1/month, 1-3/month, 1/ week, 2-4/week, 5-6/week, 1/day, 2-3/day, or >3/day)
Apple (1 unit)
Avocado (ration)
Banana (1 unit)
Berries (ration)
Kiwi (1 unit)
Melon (ration)
Olives (ration)
Orange (1 unit)
Peaches (1 unit)
Pineapple (ration)
Strawberries (ration)

Cereals (please select in each case: <1/month, 1-3/month, 1/ week, 2-4/week, 5-6/week, 1/day, 2-3/day, or >3/day)
Breakfast cereals (ration)
Pasta (ration)
White bread (ration)
Whole bread (ration)

Dairy (please select in each case: <1/month, 1-3/month, 1/ week, 2-4/week, 5-6/week, 1/day, 2-3/day, or >3/day)
Butter (ration)

Other cheese (ration)
White cheese (ration)
Yogurt (1 unit)

Milk (please write the number of glasses per day, and indicate if it is mainly whole, semi-skimmed, or skimmed)

Miscellanea (please select in each case: <1/month, 1-3/month, 1/ week, 2-4/week, 5-6/week, 1/day, 2-3/day, or >3/day)
Baked goods (ration)
Bakery (ration)
Cakes (ration)
Chocolate (ration)
Cookies (3-4 units)
Eggs (2 units)
Fritters (ration)
Mayonnaise (ration)
Nuts (ration)
Sugar (1 dose)
